# A Cross Section Study to Determine the Prevalence of Antibodies against HIV Infection among Hepatitis B and C Infected Individuals

**DOI:** 10.3390/ijerph13030314

**Published:** 2016-03-11

**Authors:** Geane L. Flores, Adilson J. de Almeida, Juliana C. Miguel, Helena M. Cruz, Moyra M. Portilho, Letícia de P. Scalioni, Vanessa A. Marques, Lia Laura Lewis-Ximenez, Elisabeth Lampe, Livia Melo Villar

**Affiliations:** Viral Hepatitis Laboratory, Oswaldo Cruz Institute, Oswaldo Cruz Foundation (FIOCRUZ), Rio de Janeiro 210360-040, Brazil; geane@ioc.fiocruz.br (G.L.F.); adilsonjoal@ioc.fiocruz.br (A.J.d.A.); julicm@ioc.fiocruz.br (J.C.M.); h.medina@ioc.fiocruz.br (H.M.C.); moyramp@ioc.fiocruz.br (M.M.P.); lescali@ioc.fiocruz.br (L.d.P.S.); vmarques@ioc.fiocruz.br (V.A.M.); llewis@ioc.fiocruz.br (L.L.L.-X.); elampe@ioc.fiocruz.br (E.L.)

**Keywords:** human immunodeficiency virus, hepatitis C virus, hepatitis B virus, prevalence

## Abstract

(1) *Background*: There are limited data regarding human immunodeficiency virus (HIV) prevalence among hepatitis B virus (HBV) or hepatitis C virus (HCV) infected individuals. The aim of this cross-sectional study is to determine the prevalence of HBV and HCV infection among HIV individuals; (2) *Methods*: A total of 409 patients (126 HBV+ and 283 HCV+) referred to the Brazilian Reference Laboratory for Viral Hepatitis from 2010 to 2013 donated serum samples. Anti-HIV, HBsAg, anti-HBc, anti-HBs, anti-HBcIgM, anti-HBe, HBeAg, and anti-HCV antibodies were measured, and anti-HCV positive samples were tested for viral RNA and genotype; (3) *Results*: The anti-HIV antibody prevalence was 10.31% and 4.59% among HBV+ and HCV+ patients, respectively. The HCV mean (SD) viral load was log 5.14 ± 1.64 IU/mL, and genotype I was most prevalent (163/283). Anti-HBs and anti-HBc were detected in 40% and 26% of HCV+ individuals, respectively. Among the HBV+ population, the presence of anti-HIV antibodies was associated with male gender, marital status (married), tattoo, sexual orientation, sexual practices (oral sex and anal sex), history of sexually transmitted diseases (STDs), history of viral hepatitis treatment, and a sexual partner with hepatitis or HIV. For the HCV+ group, the presence of anti-HIV antibodies was associated with female gender, marital status (married), anal intercourse, previous history of STDs, and number of sexual partners; (4) *Conclusion*: A high prevalence of anti-HIV antibodies was found among individuals with HBV and HCV, showing the importance of education programmes towards HIV infection among HBV- and HCV-infected individuals.

## 1. Introduction

Human immunodeficiency virus (HIV), hepatitis B virus (HBV), and hepatitis C virus (HCV) are major public health issues and share parenteral, sexual, and vertical routes as modes of transmission. Consequently, HBV or HCV infection is common among individuals with HIV/AIDS, especially regarding those who are intravenous drug users (IDUs) because these individuals often share contaminated drug injecting paraphernalia such as needles/syringes [1,2].

Worldwide, there are 240 million people chronically infected with HBV, 130–150 million chronic HCV cases, and 35 million people living with HIV/AIDS [3,4,5]. In Latin America, 1.5 million children and adults live with HIV [6], 7–12 million individuals are infected with HBV, and 7–9 million adults are anti-HCV-positive [7]. In Latin America, the majority of HIV cases (75%) are concentrated in five countries: Argentina, Brazil, Colombia, Mexico and Venezuela [8]. Chile has an HIV prevalence of 1.7%, and Brazil and Mexico together have approximately 4 million people with HCV [9,10]. In Brazil, the HIV prevalence is 0.6% [11], the HCV prevalence is 1.38% [12], and the anti-HBc prevalence varies from 3.8% in the Federal District to 5.5% in the Northeast region of country [13]. Studies conducted among specific groups, such as military personnel, children and beauticians, observed a low prevalence of HBV and HCV infection in Rio de Janeiro [14,15,16].

HIV infection increases mortality and morbidity in HBV and HCV patients. A more rapid progression of liver fibrosis to cirrhosis and hepatocellular carcinoma is more common among HIV-HBV or -HCV coinfection when these individuals are not treated [1,2]. Due to the epidemiological and clinical relevance of these coinfections, it is important to identify these subjects to give them the appropriate antiviral therapy. In addition, studies to determine the prevalence of HIV, HBV, and HCV in developing countries could help public health authorities to plan education programmes to reduce the morbidity and mortality of these infections.

Studies to determine the risk factors for HBV and HCV infection among HIV-infected individuals are common [17,18,19,20,21,22], but few studies have been conducted to determine the HIV prevalence and related risk factors among the HBV- or HCV-infected population, particularly in Latin America [23]. This study aims to investigate the seroprevalence of HIV infection in hepatitis B- and C-infected individuals referred to the Viral Hepatitis Ambulatory of the Oswaldo Cruz Institute Oswaldo Cruz Foundation (FIOCRUZ), Rio de Janeiro, Brazil, as well as risk factors associated with these infections.

## 2. Experimental Section

### 2.1. Study Design

This is a retrospective cross-sectional study among HBV- and HCV-infected individuals referred to the Viral Hepatitis Ambulatory clinic in Rio de Janeiro, Brazil. It is a one of the reference centres for hepatitis in the state of Rio de Janeiro and in Brazil. This centre receives persons suspected to be infected with viral hepatitis, including acute and chronic cases and their contacts.

In this study, a convenience sampling method was used. This is a non-probability-based sampling technique where subjects are selected because of their convenient accessibility and proximity to the researcher.

From 2010 to 2013 (period of the study), around 1000 individuals were referred to in the center. Based on the inclusion criteria, a total of 500 individuals were potentially eligible to participate in the study, 435 patients were contacted for eligibility, 420 were confirmed to be eligible (+number of HBV/HCV co-infected individuals excluded), and 409 individuals were included and analysed in this study.

Demographic and risk factor data were obtained from HBV- and HCV-infected individuals. Based on the inclusion criteria, individuals aged more than 18 years old and having a previous reactive result for HBsAg (HBV group) or reactive results for both anti-HCV/HCV-RNA (HCV group) were selected. HBV-HCV coinfected individuals were excluded from the study. Interviews were conducted in person using a structured standard questionnaire to obtain information regarding the socio-demographic characteristics, history of jaundice, blood transfusion, use of parenteral illicit/recreational drugs, as well as sexual, family, and social histories. Questionnaires were administered before sample collection, and the practices used were the current practices at the time of the interview.

All subjects gave their informed consent for inclusion before they participated in the study. The study was conducted in accordance with the Declaration of Helsinki, and the protocol was approved by the Fiocruz Ethics Committee under the number 22912113.5.0000.5248.

### 2.2. Laboratory Investigations

Blood samples were obtained by venipuncture using 10 mL vacutainer tubes (BD, Plymouth, NJ, USA) and clotted. All serum samples were stored at −20 °C until analysis. The serum samples were tested for the presence of the hepatitis B surface antigen (HBsAg), as well as antibodies to HBV core (anti-HBc) and to HBsAg (anti-HBs), HCV (anti-HCV), and HIV (anti-HIV-1/2) using commercial enzyme immunoassays (HBsAg One, Radim, Italy; ETI-AB-COREK PLUS; Diasorin, Sluggia, Italy; ETI-AB-AUK, Diasorin, Sluggia, Italy; Murex Anti-HCV Version 4.0, Diasorin, Sluggia Italy; Vironostika HIV Uni-Form II Ag/Ab, Bio-Merieux, Boxtel, The Netherlands), in accordance with the manufacturer’s instructions. HBsAg-positive samples were also tested for anti-HBc IgM, anti-HBe and HBeAg (ETI-CORE-TG MK PLUS, ETI-AB-EBK, Diasorin, Sluggia, Italy; ANTI-HBE ELECSYS, Roche, Indianapolis, IN, USA), in accordance with the manufacturer’s recommendations. Reactive tests were repeated at least once to avoid false positive results. All serological tests used for the detection of viral hepatitis and HIV markers presented estimated sensitivity and specificity of greater than 99%, as indicated by the manufacturer’s manual.

Anti-HCV reactive samples were submitted to real time PCR (Cobas Taqman HCV 2.0, Roche, Branchburg, NJ. USA), which has a dynamic range of linear quantification of 20 to 1.7 × 10^8^ IU/mL, and samples with detectable HCV RNA were also genotyped by INNOLIPA (Versant HCV Genotype Assay—LiPA, Bayer, Erlangen, Germany) [24].

### 2.3. Data Analysis

The data collected were expressed as the mean ± standard deviation (SD) and frequencies. The prevalence rate was calculated for anti-HIV in the studied population.

Descriptive statistics were generated for the responses (according to the questionnaire items), and Fisher’s exact test (for small numbers) and a chi-squared test for trends were used to compare the dichotomic and nominal variables, respectively, according to anti-HIV status. Variables selected for their relevance and statistical significance (*p* < 0.20) in the bivariate analysis were entered into the logistic regression using the enter method. The 95% confidence intervals (95% CI) of the estimated odds ratio (OR) were also calculated. The results were considered statistically significant in the multivariate analysis if the *p*-value was <0.05. All analyses were performed with the Statistical Package for the Social Sciences (SPSS for Windows, release 20.0; SPSS, Inc., Chicago, IL, USA).

## 3. Results

### 3.1. Studied Population

A total of 409 individuals were recruited during the study period, of whom 126 were HBV-infected and 283 were HCV-infected individuals. Table 1 summarizes the socio-demographic characteristics and risk factors of the study population. The overall mean age ± SD was 37.3 ± 9.5 years and 55.4 ± 10.6 years for HBV and HCV individuals, respectively. Most of the individuals were more than 37 years old, with a predominance of males (58.7%) and females (63.6%) among HBV individuals and HCV individuals, respectively.

Regarding the socio-demographic characteristics, most of the individuals were not married and received up to US$985.00 dollars per month (69.9% in the HCV group and 49.2% in the HBV group). Most of the HBV+ individuals attended up through primary or secondary school, and most of the HCV individuals attended up through pre- or primary school.

A history of jaundice was common in both groups, but a history of antiviral interferon-based therapy was more common among HCV+ individuals. Blood transfusion was more common among HCV+ individuals (50.5%), and 87.4% were transfused before 1994. In both groups, only a few individuals reported haemodialysis and acupuncture. Intravenous medicine administration history was more frequent in the HCV+ group (66.8%). The majority of the individuals in both groups reported dental treatment procedures and earrings/piercings, but few individuals reported sharing toothbrushes and razors/blades.

Less than 15% of the HBV- and HCV-infected individuals reported tattooing. Among the HCV+ individuals, 193 (68.2%) reported visits to manicurists, but 133 of them did not use their own personal care items such as nail clippers or scissors, and 81 of them reported being handled with sterilised items. Among HBV+ individuals, 72 (57.1%) reported visiting manicurists, where 56 did not use their own personal care items, and 23 reported that sterilised items were used.

Consumption of alcohol was more frequent among HBV+ individuals, but a history of illicit narcotic substances was more common among HCV+ individuals. In the latter group, 24 (68.6%) rarely used these substances, while nine (25.7%) individuals reported frequent intake. In the HBV+ group, eight individuals reported illicit narcotic substances, and four (50%) of them had used cocaine.

Regarding sexual behaviour, most of the individuals in both groups were heterosexual (HBV+ group, *n* = 104; HCV group, *n* = 262), and 8.7% and 5.3% of the HBV- and HCV-infected individuals, respectively, had a sexual partner with HIV or viral hepatitis. Among HCV+ individuals, 170 had a regular sexual partner, 80 had less than five partners per year and 10 had five or more partners per year. Among the HBV+ individuals, 88 had regular partners and 18 had less than five partners per year. Regarding condom usage, 48 HCV+ and 37 HBV+ individuals reported always using condoms, while 135 HCV+ and 45 HBV+ individuals reported never using condom during sexual intercourse. Few subjects reported intradomiciliary contact with other individuals with hepatitis or HIV or previous STD in both groups. The practice of oral sex was more frequent among HBV-infected individuals, while anal sex was more common among HCV-infected individuals.

### 3.2. Prevalence of Anti-HIV-1/2 Antibodies and Relationship to Demographic and Risk Behaviour among HBV and HCV Individuals

The prevalence of anti-HIV-1/2 antibodies was 10.31%, confidence interval (CI) 95%: 5.1–15.5 (13/126) and 4.59% (CI 95%: 2.1–7.0) (13/283) among HBV- and HCV-infected individuals, respectively (Table 1).

Among HBV-infected individuals, 119 were anti-HBc reactive, 16 were anti-HBc IgM reactive, 89 were anti-HBe reactive, and 21 were HBeAg reactive. Among HCV-infected individuals, 73 were anti-HBc reactive and 107 were anti-HBs reactive. The HCV-RNA viral load was log 5.14 ± 1.64 IU/mL, and 180/283 HCV-RNA positive samples were genotyped (HCV-1, *n* = 163; HCV-2, *n* = 1, HCV-3, *n* = 14; and HCV-5, *n* = 2).

In bivariate analysis, sexual orientation, number of sexual partners, practice of oral sex, practice of anal sex, previous history of STDs, and having a partner with hepatitis or HIV were found to be statistically significant when comparing HBV-monoinfected with HIV/HBV-infected individuals (Table 2). However, no variable was significant in multivariate analysis.

Regarding bivariate analysis in the HCV- and HIV/HCV groups, the following variables were statistically significant: the practice of anal sex and a previous history of STDs (Table 3). In multivariate analysis, female gender and a previous history of STDs were found to be statistically significant.

## 4. Discussion

This study shows a high prevalence of HIV antibodies among HBV- and HCV-infected individuals evaluated at the Viral Hepatitis Ambulatory clinic in Rio de Janeiro, Brazil. Worldwide, there are 240 million people chronically infected with HBV, 130–150 million chronic HCV cases and 35 million people living with HIV/AIDS [3,4,5]. Most studies have evaluated HBV and HCV prevalence among HIV-infected individuals, where a low prevalence of HBV and HCV markers has been found in Brazil (1% for HBsAg and 1.6% for HCV) [17], Colombia (2.1% for HBsAg and 0.8% for HCV), Nigeria (7.9% for HBsAg and 2.3% for HCV) and India (2.6% for HBsAg and 1.7% for HCV) [18,19,20]. On the other hand, a high prevalence of HBV and HCV was found in African countries such as Tanzania (17.3% for HBsAg and 18.1% for HCV), Gambia (12.2% for HBsAg) and Cote D’Ivoire (13.4% for HBsAg) [20,21,22].

Socio-demographic characteristics and risk factors were investigated in the present study. Family income and education level were relatively low, similar to that observed in studies conducted among HIV/HCV coinfected patients in Brazil [25]. The poverty variable has been evaluated in conjunction with race and stigma in relation to the risk of HIV infection, and the data have demonstrated that these three variables act together to increase the risk of HIV infection [26].

Some risk factors for HIV acquisition were common in both groups of HBV+ and HCV+ individuals, such as a history of intravenous medicine administration, dental procedures, earrings/piercings, and having manicures and pedicures. Recently, sharing nonsterilised manicure/pedicure instruments was described as a possible route of HIV-1 transmission [27]. On the other hand, consumption of alcohol was more frequent among HBV+ individuals, but a history of illicit narcotic substances was more common among HCV+ individuals. Illicit drug usage has been associated with a higher risk of HIV acquisition, likely due to sharing drug paraphernalia [28]. Regarding sexual behaviour, most of the individuals were heterosexual, reported regular sexual partners and never used condoms during sexual encounters. Thus, the risky sexual behaviour observed in these individuals could also contribute to the risk of HIV infection in this group. Although a regular partner is a factor that contributes to reducing HIV and viral hepatitis transmission, the absence of condom usage is related to a high frequency of these infections, as demonstrated in other studies [29,30].

In the present study, almost 40% of the HCV+ individuals presented HBV immunity (anti-HBs reactive sera), and 26% showed serological evidence indicating past HBV infection (anti-HBc reactive sera). The HBV immunity rate was lower than that observed among HIV/HCV-infected individuals from China [31]. Most of the HCV+ individuals had a high viral load compared with a previous study conducted among HIV/HCV-infected individuals [32]. HCV genotype 1 was the most prevalent, similar to that observed among HIV/HCV-coinfected and HCV-monoinfected individuals in Brazil [12,33].

HIV prevalence was associated in the bivariate analysis with sexual orientation, the practice of anal sex, a previous history of STDs and the number of sexual partners among HCV+ and HBV+ individuals. Previous studies showed that having multiple sexual partners, practicing unsafe sex, and sexual orientation are associated with a higher prevalence of HIV [30,31,34,35]. This may be due to the risk behaviours associated with these practices, which increases the likelihood of HIV infection.

Among the HBV+ group, according to the bivariate analysis, male gender, a history of hepatitis treatment, tattooing, the practice of oral sex and partners with viral hepatitis or HIV were also associated with HIV positivity. Kapembwa *et al.* [35] observed that vaccination history and tattooing were common among HIV/HBV-coinfected individuals; however, the possible reasons for this association were not demonstrated. In Brazil, there are technical standards for the proper use of needles in tattoo procedures [36], and immunization against HBV is available in public health settings for individuals until 49 years and some risk groups, such as HIV-infected individuals [37].

Among the HCV+ group, HIV positivity was associated with female gender and a history of STDs in multivariate analysis. Because STDs share the same mode of transmission as HIV, the presence of other diseases could increase the risk of HIV acquisition [38,39]. A previous study showed that females may be five times more likely to become infected with HIV, and this prevalence was associated with sexual behaviour such as prostitution, injection drug use and poor living conditions [40]. Furthermore, this result reflects the feminization of the HIV epidemic that has been documented in Brazil and other countries, where women represent approximately 50% of people living with HIV and may even reach the majority in certain age groups [41].

This study presents some limitations. Because the study design was cross-sectional, a causal relationship between the time of exposure and subsequent infection could not be established. The recruitment of the population using convenience sampling could not allow for sample calculation. It remains possible that biases in the study population due to convenience sampling may have compromised some results. Another limitation of this study could be the investigation of sexual practices and drug abuse because the patients may not report true answers in an in-person interview.

## 5. Conclusions

A high prevalence of HIV antibodies was found among HBV- and HCV-infected individuals in the present study. Due to the possibility of transmission of these viruses by the parenteral route, HIV prevalence could be high in other settings, reinforcing the need for education programmes focusing on HIV infection, particularly among HBV- and HCV-infected individuals.

## Figures and Tables

**Table 1:** Sociodemographic clinical and variables studied in HBV- and HCV-infected individuals of the study.

Characteristics	HBsAg Reactive (*n* = 126)	Anti-HCV Reactive (*n* = 283)
**Age group; *n* (%)**	–	–
18–19 years	16 (12.7)	0 (0.0)
20–39 years	39 (30.9)	27 (9.5)
40–59 years	51 (40.5)	147 (51.9)
>59 years	20 (15.9)	109 (38.5)
**Gender; *n* (%)**	–	–
Male	74 (58.7)	103 (36.4)
Female	52 (41.3)	180 (63.6)
**Anti-HIV status; *n* (%)**	–	–
Reactive	13 (10.3) (IC 95%: 5.1–15.5)	13 (4.6) (IC 95%: 2.1–7.0)
Non-reactive	113 (89.7)	270 (95.4)
**Marital status; *n* (%)**	–	–
Married	61 (48.4)	125 (44.2)
Not married	65 (51.6)	158 (55.8)
**Education; *n* (%)**	–	–
Illiterate	1 (0.8)	6 (2.1)
Pre Primary Education	37 (29.4)	88 (31.1)
Primary Education	25 (19.8)	67 (23.7)
Secondary Education	39 (31.0)	81 (28.6)
College	24 (19.0)	41 (14.5)
**Monthly income; *n* (%)**	–	–
≤US$985.00	62 (42.2)	198 (69.9)
>US$985.00	37 (29.4)	75 (26.5)
**Number of sexual partners**	–	–
Regular Partner	88 (69.8)	170 (60)
Less than 5 per year	18 (14.3)	80 (28.3)
More than 5 per year	2 (1.8)	10 (3.5)
**Jaundice; *n* (%)**	87 (69.0)	203 (71.7)
**Hepatitis treatment; *n* (%)**	17 (13.5)	96 (33.9)
**Previous blood transfusion; *n* (%)**	16 (12.7)	143 (50.5)
**Haemodialysis; *n* (%)**	1 (0.8)	9 (3.2)
**Acupuncture; *n* (%)**	7 (5.5)	40 (14.1)
**Intravenous medicine; *n* (%)**	62 (49.2)	189 (66.8)
**Dental procedure; *n* (%)**	106 (84.1)	259 (91.5)
**Pierced ear; *n* (%)**	62 (49.2)	163 (57.6)
**Sharing blades; *n* (%)**	39 (31.0)	79 (27.9)
**Sharing instruments such as toothbrush; *n* (%)**	15 (11.9)	28 (9.9)
**Tattoo; *n* (%)**	16 (12.7)	29 (10.2)
**Manicure; *n* (%)**	72 (57.1)	193 (68.2)
**Consumption of alcohol at least once per week; *n* (%)**	35 (27.8)	50 (17.7)
**Illicit narcotic substances (injectable and inhalable)**	8 (6.3)	35 (12.4)
**Sexual partner with HIV or viral hepatitis; *n* (%)**	11 (8.7)	15 (5.3)
**Intradomiciliar contact with viral hepatitis or HIV; *n* (%)**	38 (30.2)	36 (12.7)
**Previous sexually transmitted infection; *n* (%)**	24 (19.0)	57 (20.1)
**Practice of oral sex; *n* (%)**	52 (41.3)	108 (38.2)
**Practice of anal sex; *n* (%)**	34 (27.0)	85 (30.0)

**Table 2:** Analysis of variables studied for HIV among HBV-infected individuals (*n* = 126).

Variable	HBV Monoinfected *n* = 113	HBV/HIV Coinfected *n* = 13	Bivariate Analysis Unadjusted Odds Ratio (95% CI)	Bivariate Analysis *p-*Value
**Gender, male**	63 (55.7%)	11 (84.6%)	4.365 (0.9246–20.609)	0.0716
**Married**	57 (50.4%)	04 (30.8%)	0.3070 (0.086–1.096)	0.0672
**History of Hepatitis treatment**	14 (12.4%)	03 (23.1%)	4.125 (0.8312–20.472)	0.0980
**Tattoo**	12 (10.6%)	04 (30.8%)	8.000 (2.391–26.765)	0.0013
Sexual Orientation *	–	–	–	–
Heterosexual	101 (89.4%)	03 (23.1%)	54.097	0.000
Homosexual	04 (3.5%)	06 (46.1%)		
Bisexual	00 (0.0%)	02 (15.4%)		
Number of sexual partners *	–	–	–	–
Regular Partner	83 (73.4%)	05 (38.5%)	9.944	0.019
<5 partners per year	13 (11.5%)	05 (38.5%)		
≥5 partners per year	02 (1.8%)	00 (0.0%)		
**Practice of oral sex**	41 (36.3%)	11 (84.6%)	21.892 (1.247–384.35)	0.002
**Practice of anal sex**	25 (22.1%)	09 (69.3%)	9.540 (1.917–47.473)	0.002
**Previous history of sexually transmitted infections**	17 (15.0%)	07 (53.8%)	12.353 (2.901–52.598)	0.000
**Partner with hepatitis or HIV**	7 (6.2%)	4 (30.7%)	13.143 (2.432–71.030)	0.005

No variable was significant in multivariate analysis. * Statistical analysis was performed using a chi-square test for trends. For other variables, Fisher’s exact test was used.

**Table 3:** Analysis of variables studied for HIV among HCV-infected individuals (*n* = 283).

Variable	HCV Monoinfected *n* = 270	HCV/HIV Coinfected *n* = 13	Bivariate Analysis Unadjusted Odds Ratio (95% CI)	Bivariate Analysis *p-*Value	Multivariate Analysis Unadjusted Odds Ratio (95% CI)	*p-*Value
**Gender, female**	169 (62.6%)	11 (84.6%)	0.3042 (0.0667-1.401)	0.143	10.824 (1.631–71.831)	0.014
Marital status, married *	122 (45.2%)	03 (23.1%)	4.736	0.192	1.481 (0.742–2.954)	0.266
Sexual Orientation *	–	–	–	–	–	–
Heterosexual	251 (93.0%)	11 (84.6%)	4.203	0.122	1.046 (0.154–7.120)	0.964
Homosexual	03 (1.1%)	01 (7.7%)				
Bisexual	04 (1.5%)	00 (0.0%)				
**Practice of anal sex**	76 (28.1%)	09 (69.2%)	5.063 (1.512-16.955)	0.011	3.695 (0.739–18.470)	0.111
**Previous history of sexually transmitted infections**	47 (17.4%)	10 (76.9%)	14.610 (3.868-55.178)	0.000	33.338 (5.462–203.492)	0.000
Number of sexual partners *	–	–	–	–	–	–
Regular Partner	165 (61.1%)	05 (38.5%)	7.970	0.093	1.297 (0.766–2.198)	0.333
<5 partner per year	73 (27.0%)	07 (53.8%)				
≥5 partners per year	09 (3.3%)	01 (7.7%)				

* Statistical analysis was performed using a chi-square test for trends. For other variables, Fisher’s exact test was used.
